# Completion rate of physician orders for life-sustaining treatment for patients with metastatic or recurrent cancer: a preliminary, cross-sectional study

**DOI:** 10.1186/s12904-019-0475-9

**Published:** 2019-10-22

**Authors:** Ju Won Kim, Jung Yoon Choi, Won Jin Jang, Yoon Ji Choi, Youn Seon Choi, Sang Won Shin, Yeul Hong Kim, Kyong Hwa Park

**Affiliations:** 1 Division of Oncology/Hematology, Department of Internal medicine, Korea University Anam Hospital, 73 Goryeodae-ro Seongbuk-gu, Seoul, 02841 South Korea; 2 Department of Family Medicine, Korea University Guro Hospital, 97 Guro-dong Gil, Guro-gu, Seoul, 08308 South Korea

**Keywords:** Physician orders for life-sustaining treatment, End-of-life care, Advance directives

## Abstract

**Background:**

“End of life” is a difficult topic of conversation in East Asian cultures, even among patients and doctors who share a good rapport. In 2016, the Hospice, Palliative Care, and Life-Sustaining Treatment Decision-Making Act, which took the form of “Physician Orders for Life-Sustaining Treatment,” was introduced in South Korea. This study was conducted to investigate the completion rate of Physician Orders for Life-Sustaining Treatment in patients with advanced cancer on the active recommendation of physicians, as well as patients’ general attitudes toward end-of-life care.

**Methods:**

We conducted a preliminary, cross-sectional descriptive survey on patients with advanced cancer. A total of 101 patients with advanced solid cancer agreed to participate in the study. The primary endpoint was the rate of completion of Physician Orders for Life-Sustaining Treatment based on a doctor’s suggestion. Written interviews were conducted to understand the perceptions and factors influencing patients’ decisions.

**Results:**

Of the 101 patients, 72 (71.3%) agreed to prepare Physician Orders for Life-Sustaining Treatment. Patients who had an educational level of high school or higher were more likely to agree to complete Physician Orders for Life-Sustaining Treatment documentation as compared to the lower educational status group. More than half of the respondents who completed Physician Orders for Life-Sustaining Treatment documentation reported that they had more than a fair understanding of “life-sustaining care” or “Physician Orders for Life-Sustaining Treatment.” Participants’ reasons for Physician Orders for Life-Sustaining Treatment completion were diverse.

**Conclusions:**

We found that highly educated patients, who understood the concept behind the policy well, tended to accept Physician Orders for Life-Sustaining Treatment without hesitation. Better education, information shared through the media, and conversations with health care providers might improve understanding of Physician Orders for Life-Sustaining Treatment in patients with cancer.

## Background

Talking about death and dying is one of the key steps in developing an advance care plan (ACP) for patients with cancer. However, end of life (EOL) is a sensitive topic of conversation even between patients and health care providers who share a good rapport. In addition to the sensitivity of the topic, the appropriate process and timing are controversial, making conflicts in the medical field inevitable.

In South Korea, as the demand for establishing a legal basis for the EOL decision-making process increased, the Hospice, Palliative Care, and Life-Sustaining Treatment Decision-Making Act passed the plenary session of the National Assembly in January 2016. After a three-month pilot period, the Act was fully implemented on February 4, 2018. Under this Act, those who meet the requirements can state their intentions regarding life-sustaining medical care through the Physician Orders for Life-Sustaining Treatment (POLST) document [1]. POLST is a part of an ACP with advance directives (AD) and is written by a doctor based on the patient’s wishes at the terminal stage (Additional file 2: Figure S1).

Along with geriatric chronic diseases, cancer is cited as a major cause of mortality in South Korea. This is because almost all kinds of cancer ultimately cause multi-organ failure with metastases, which is often difficult to revive in spite of active treatment. Along with the context that there is lack of palliative care specialist in South Korea, the policy around ACT was discussed mainly among physicians who treat cancers. Moreover, medical oncologists were the most important instructor to patients in the clinic.

Nevertheless, the sociocultural situation in East Asian countries such as South Korea is still such that discussions of ACP, including EOL, tend to be uncomfortable [2, 3]. Additionally, proposals to prepare for dying well are still taboo. Decisions regarding the dying process are more likely to be a reflection of complex familial dynamics than the patient’s free will [4]. To achieve the purpose of the legislation, it is essential that not only medical staff and caregivers but also patients themselves be fully aware of ACP policies.

However, there are no studies on the real-world barriers to EOL care and POLST decision-making among patients with cancer, and there is a lack of nationwide surveys on patients’ perceptions. We, therefore, conducted this study to investigate the completion rate of POLST in patients with advanced cancer when it is actively suggested by their physicians, and to examine patients’ general attitudes toward EOL.

## Methods

### Study protocol

This was a single-center, preliminary, cross-sectional study conducted from June 2018 to January 2019 at the Korea University Anam Hospital Cancer Center. Patients were offered POLST at physicians’ discretion if they were considered to be nearing EOL based on their medical progress. The doctors defined eligible patients based on remnant organ function, poor general condition, which was defined as Eastern Cooperation Oncology Group grade 3 or 4, and the lack of available treatment choice. Three medical oncologists directly provided an outline of POLST to their patients who were eligible for the study. After a thorough discussion, patients were asked to decide whether they would prepare the document that day. After they had made their decision, patients were asked to complete questionnaires in a protected space for up to 30 min, regardless of whether or not they decided to complete POLST documentation. Participants filled out and submitted the questionnaire on the spot. Written informed consent was obtained from all participants and the study was approved by the Institutional Review Board (IRB) of Korea University Hospital (IRB no. 2018AN0152).

### Study participants

This study aimed to investigate the POLST completion rate in patients with recurrent or metastatic cancer who were currently undergoing treatment. Only patients over 19 years of age who could understand the purpose of the study and provide informed consent were included. Patients who could not understand the Korean questionnaire were excluded. There was no distinction made on the basis of type of cancer.

### POLST in South Korea and survey questionnaire

In South Korea, the Hospice, Palliative Care, and Life-Sustaining Treatment Decision-Making Act introduced AD and POLST as legal forms of ACP in 2016. The South Korean POLST documentation contains basic information on the patient and doctor and an indication of the decision to refuse life-sustaining treatment. Cardiopulmonary resuscitation (CPR), artificial respiration through tracheal intubation, hemodialysis, and anti-cancer therapy are represented as optional life-sustaining medical services, and patients can choose whether or not to implement any of them in their last days of life. As a part of ACP, the POLST document may also include choice of hospice palliative care. After the patient has confirmed their choice by signing the document, it is registered in the central government system and preserved for 10 years.

We conducted a survey questionnaire to identify factors associated with the completion rate of POLST. The questionnaires were reviewed and confirmed by multiple medical staff members, including medical oncologists and family physicians. The survey questionnaire requested the following: (1) demographic information: age, gender, religion, level of education, and monthly income; (2) self-reported level of understanding of life-sustaining treatment and POLST (very familiar, familiar, fair understanding, not familiar, never heard of it); (3) major sources of information on life-sustaining treatment and POLST; (4) reason for choosing (or refusing) to complete POLST documentation; (5) person with whom they discussed their EOL care; (6) optimal timing for discussing ACP; (7) ideal place to meet their EOL; and (8) whether or not they wanted to avail the services of a hospice care center and why.

### Statistical analysis

The primary endpoint of this study was the POLST completion rate in patients with metastatic or recurrent cancer who were undergoing active anti-cancer treatment. Secondary outcomes were factors related to POLST completion rate and various aspects of practical EOL care.

If the questionnaire was not answered completely, only the collected data were analyzed. The questions that were not responded to were treated as missing data, and no specific alternative was used. Multiple responses were allowed depending on the type of question, and these were calculated separately for each question.

The chi-square test was used to examine associations between patients’ demographic characteristics, degree of awareness of the system, and POLST completion. All reported *p*-values were two sided, and p-values < 0.05 were considered significant. Statistical analysis was conducted using SPSS version 24.0.

## Results

### Study population

**Fig. 1:**
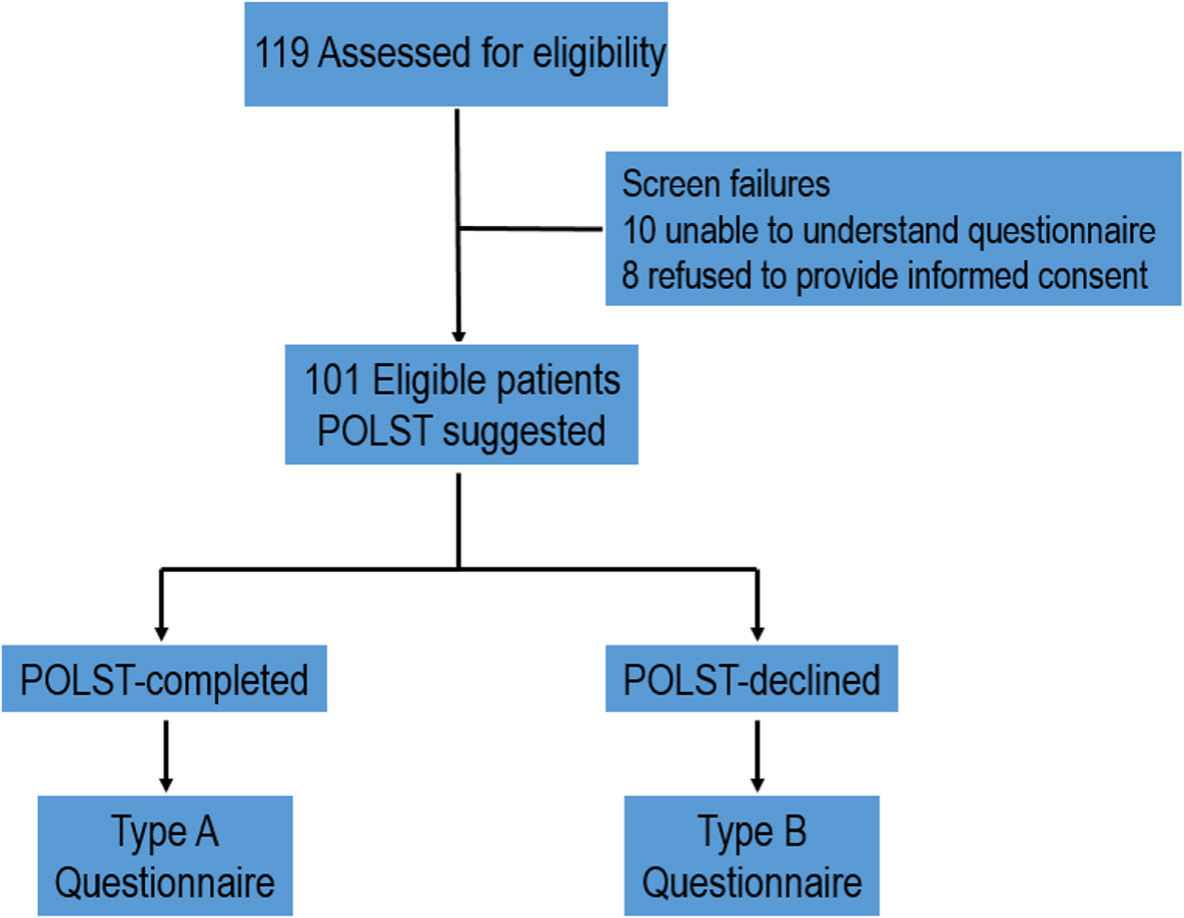
Schematic representation of the study population

A total of 119 patients were offered POLST, and 101 were eligible to participate in this study; a schematic depiction of the study population is presented in Fig. 1. The return rate of the questionnaires was 100%, and an analysis was conducted based on the patients’ responses. The demographic characteristics of the 101 participants are summarized in Table 1. The median age was 64 years (range 30–90 years), and the proportion of male and female participants was similar (male 53.5%, female 46.5%). Breast cancer was the most common type of cancer (33, 32.7%), followed by prostate cancer (22, 21.8%). Median time from cancer diagnosis to suggestion of POLST was 30.8 months (range 1.8–262.5), and median follow-up period by the physician who suggested POLST was 16.8 months (range 1.2–106.9). Most of the patients participated in the study during their visit to the outpatient clinic (93.1%).

**Table 1:** Demographic features of the study population (*N* = 101)

Characteristics	No. (%)
Age (years)	
31–40	3 (3.0%)
41–50	11 (10.9%)
51–60	24 (23.8%)
61–70	29 (28.7%)
71–80	27 (26.7%)
81–90	7 (6.9%)
Gender	
Male	47 (46.5%)
Female	54 (53.5%)
Cancer type	
Breast	33 (32.7%)
Prostate	22 (21.8%)
Lung	2 (2.0%)
Gastrointestinal	27 (26.7%)
Genitourinary (except prostate)	8 (7.9%)
Hepatobiliary	5 (5.0%)
Others ^a^	4 (4.0%)
Religion	
No religion	43 (42.6%)
Follow a religion ^b^	57 (56.4%)
Unknown	1 (1.0%)
Educational status	
High school or below	40 (39.6%)
High school or above	61 (60.4%)
Monthly income	
Less than $2000	77 (76.2%)
More than $2000	20 (19.8%)
Unknown	4 (4.0%)
Place of POLST suggestion	
Outpatient clinic	94 (93.1%)
General ward	7 (6.9%)

^a^ Others: 3 sarcomas, 1 melanoma

^b^ 21 Christianity, 8 Catholicism, 23 Buddhism, 5 Other

### POLST completion rate and reason for the decision

**Fig. 2:**
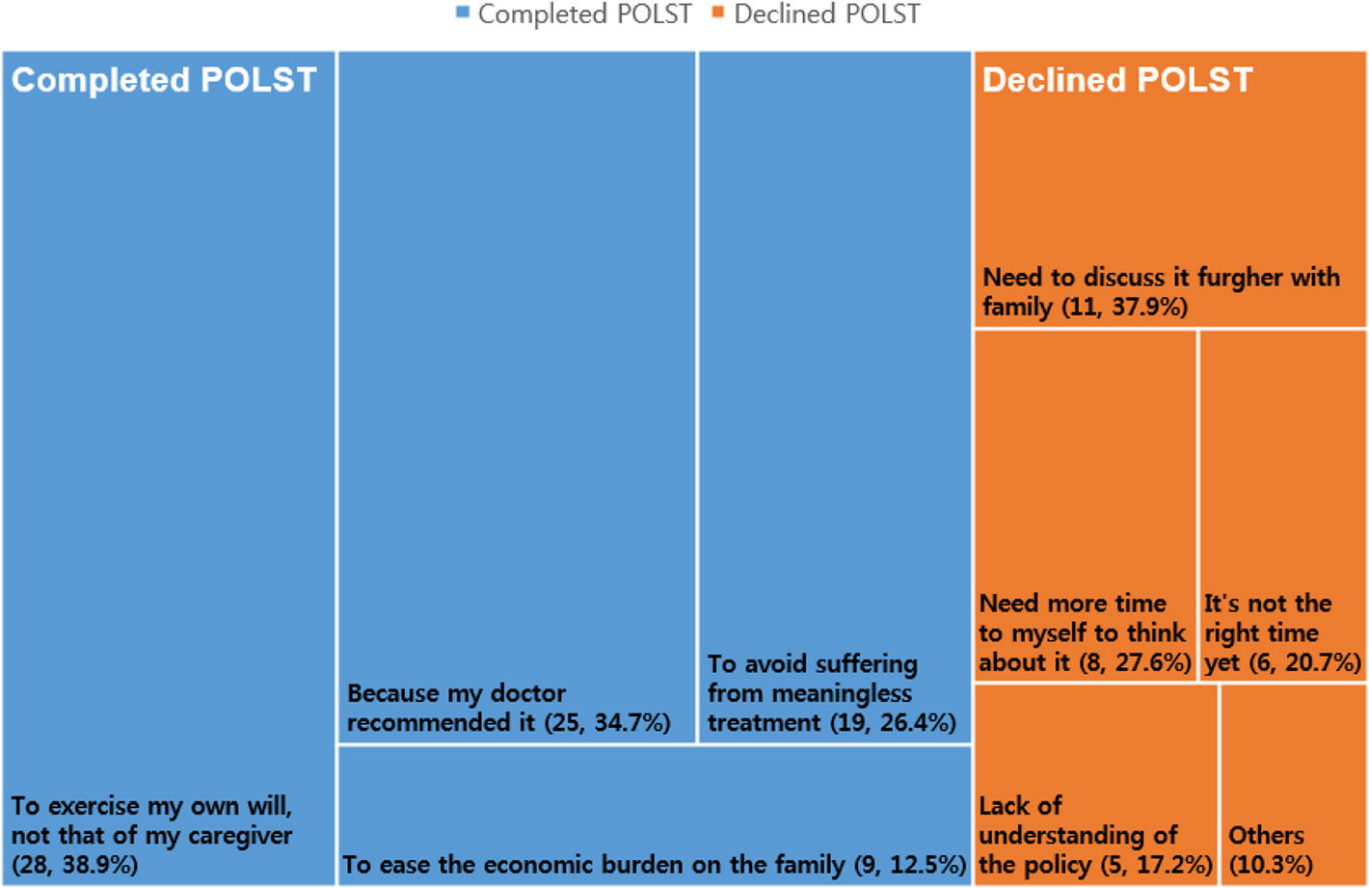
POLST completion rate and reasons for completing or declining to complete POLST. The total POLST completion rate was 71.3%. A larger area is indicative of a higher response rate. “To exercise my own will, not that of my caregiver” was the most popular reason for completing POLST, and “need to discuss it further with family” was the most common reason for declining POLST

Of the 101 patients, 72 (71.3%) agreed to sign the POLST form and registered the document on the same day. Out of the 72 patients who completed POLST documentation, 49 (67.1%) excluded CPR, artificial respiration, and hemodialysis, but wanted to continue with chemotherapy. Further, 21 patients (29.2%) did not want any kind of life-sustaining treatment, and two patients (2.8%) who were in the fifth stage of chronic kidney disease at the time of signing the POLST form wanted to suspend only CPR and artificial respiration. In total, CPR and artificial respiration were declined by all participants who prepared POLST, and hemodialysis was refused by 97.2%. However, the suspension of chemotherapy was requested by only 31.9% of patients. The questionnaires provided to each group presented four choices for the reason they decided (not) to prepare POLST. The results and the proportion of each answer are visualized in Fig. 2. The most common reason for preparing POLST was “to exercise my own will, not that of my caregiver” (28, 38.9%), followed by “because my doctor recommended it” (25, 34.7%). The two major reasons for refusal were “need to discuss it further with family” (11, 37.9%) and “need more time by myself to think about it” (8, 27.6%).

### POLST completion rate according to demographic features

We compared POLST completion rate between two groups stratified by demographic features. We divided patients according to their age and gender, educational status, monthly income, and whether or not they followed a religion, and then analyzed which group tended to complete POLST documentation. Mean or median values were used as the basis for dividing the two groups. Table 2 summarizes the POLST completion rate according to demographic and socioeconomic features. According to the results, younger (73.1% vs. 69.4%, *p* = 0.626) female (72.3% vs. 70.4%, *p* = 1) patients who did not follow a religion (72.1% vs. 70.2%, p = 1) and had a high educational level (82.0% vs. 56.4%, *p* = 0.007) and higher monthly income (85.0% vs. 66.2%, *p* = 0.169) showed the highest rate of POLST completion. None of the differences in POLST completion rate between the groups were significant except for educational status.

**Table 2:** POLST completion rate according to demographic features

Variable	Total (N = 101)	POLST completion	POLST non-completion	*p*-value
Age, n. (%)				0.826
64 or lower	52 (51.5%)	38 (73.1%)	14 (26.9%)	
65 or above	49 (48.5%)	34 (69.4%)	15 (30.6%)	
Gender, n. (%)				1.000
Male	54 (53.5%)	38 (70.4%)	16 (29.6%)	
Female	47 (46.5%)	34 (72.3%)	13 (27.7%)	
Education, n. (%)				0.007
High school or below	39 (38.6%)	22 (56.4%)	17 (43.6%)	
High school or above	61 (60.4%)	50 (82.0%)	11 (18.0%)	
Monthly income, n. (%)				0.169
$2000 or lower	77 (76.2%)	51 (66.2%)	26 (33.8%)	
Over $2000	20 (19.8%)	17 (85.0%)	3 (15.0%)	
Religion, No. (%)				1.000
No religion	43 (42.6%)	31 (72.1%)	12 (27.9%)	
Follow religion	57 (56.4%)	40 (70.2%)	17 (29.8%)	
Time from cancer diagnosis to POLST suggestion, n. (%)				0.172
One year or less	20 (19.8%)	17 (85.0%)	3 (15.0%)	
More than one year	81 (80.2%)	55 (67.9%)	29 (35.8%)	
Follow-up period by physician who suggested POLST, n. (%)				0.826
One year or less	40 (39.6%)	28 (70.0%)	12 (30.0%)	
More than one year	61 (60.4%)	44 (72.1%)	17 (27.9%)	

### Comparison of awareness between POLST-completion and non-completion groups

The perception of life-sustaining treatment and POLST was investigated, and the results are summarized in Table 3. The understanding of both concepts was higher in the POLST-completion group, and this was statistically significant (average “EOL care” understanding score 3.32 vs. 2.68, *p* = 0.016 and “POLST” understanding score 2.99 vs. 2.34, *p* = 0.036). In both groups, conventional media like newspapers and television were the main source of information about EOL, followed by medical staff (50.0 and 31.9% in the completion group, and 48.3 and 20.7% in the non-completion group, respectively).

**Table 3:** POLST completion rate according to degree of understanding

Variable	Total (*N* = 101)	POLST- completion (*n* = 72)	POLST non-completion (*n* = 29)	*p*-value
Familiarity with “life sustaining treatment”, n. (%)				
Very familiar	9 (8.9%)	7 (9.7%)	2 (6.9%)	
Familiar	31 (30.7%)	26 (36.1%)	5 (17.2%)	
Fair understanding	35 (34.7%)	27 (37.5%)	8 (27.6%)	
Not familiar	15 (14.9%)	7 (9.7%)	8 (27.6%)	
Hardly know about it	10 (9.9%)	5 (6.9%)	5 (17.2%)	
Unaware	1 (1.0%)	0 (0.0%)	1 (3.4%)	
*Median score*^a^	3.14	3.32	2.68	0.016
Information sources ^b^				
Family or other people	6 (5.9%)	6 (8.3%)	0 (0.0%)	
Newspaper or television news	50 (49.5%)	36 (50.0%)	14 (48.3%)	
Internet	7 (6.9%)	6 (8.3%)	1 (3.4%)	
Physician	29 (28.7%)	23 (31.9%)	6 (20.7%)	
Never heard about it	11 (10.9%)	6 (8.3%)	5 (17.2%)	
Familiarity with “POLST”, n. (%)				
Very familiar	11 (10.9%)	8 (11.1%)	3 (10.3%)	
Familiar	22 (21.8%)	18 (25.0%)	4 (13.8%)	
Fair understanding	26 (25.7%)	22 (30.6%)	4 (13.8%)	
Not familiar	20 (19.8%)	13 (18.1%)	7 (24.1%)	
Hardly know about it	22 (21.8%)	11 (15.3%)	11 (37.9%)	
Unaware	0 (0.0%)	0 (0.0%)	0 (0.0%)	
*Median score*^a^	2.80	2.99	2.34	0.036
Information sources ^b^				
Family or other people	7 (6.9%)	5 (6.9%)	2 (6.9%)	
Newspaper or television news	38 (37.6%)	27 (37.5%)	11 (37.9%)	
Internet	7 (6.9%)	6 (8.3%)	1 (3.4%)	
Physician	40 (39.6%)	32 (44.4%)	8 (27.6%)	
Never heard about it	17 (16.8%)	10 (13.9%)	7 (24.1%)	

^a^ Median score of awareness when “very familiar” was scored 5 and “never heard about it” was scored 1

^b^ Multiple responses were allowed. All percentage data were presented with the percentage of cases

### Questions about EOL care

Participants were asked three questions regardless of whether or not they decided to complete POLST documentation (Additional file 1: Table S1). The decision regarding ACP was mainly taken by the patients themselves (51, 50.5%), followed by the spouse (21, 20.8%) and physicians (18, 17.8%). Almost half the participants chose “when someone is young and healthy” (50, 49.5%) as the optimal time for preparing POLST. The most suitable places to meet their EOL were “home” (35, 34.7%) and “university hospitals” (24, 23.8%).

### Willingness to use a hospice care center

The South Korean POLST recommends indicating one’s willingness to use a hospice care center. The present survey also asked about this and collected the reasons for each answer (Table 4). Interestingly, the answers recorded in the POLST documents and survey questionnaires were not perfectly concordant. When physicians asked patients whether or not they wanted to use hospice palliative care, 41 (56.9%) out of 72 said “yes.” However, when the participants were asked about this in a separate room during the questionnaire survey, seven out of 41 said they did not wish to use it. There were 21 (29.2%) patients who completed POLST documentation but did not indicate their decision regarding hospice palliative care in the legal documents, and in the questionnaire, 13 of them indicated that they would not use a hospice care center. According to the survey, the main reasons for using hospice palliative care were “to receive professional treatment for pain and symptoms” (26, 43.3%) and “to reduce the burden on caregivers” (25, 41.7%). The most common reasons for not wanting to use hospice palliative care were “I don’t think it would be particularly helpful” (18, 40.0%) and “because I want to be treated by the doctor I’ve been seeing” (17, 37.8%).

**Table 4:** Preference regarding hospice palliative care

POLST		Survey questionnaire	
Yes, I would like to use hospice palliative care, n. (%)	41 (56.9%)	Yes	34 (47.2%)
		No	7 (9.7%)
No, I would not like to use hospice palliative care, n. (%)	10 (13.9%)	Yes	1 (1.4%)
		No	9 (12.5%)
Unanswered, n. (%)	21 (29.2%)	Yes	8 (11.1%)
		No	13 (18.1%)

## Discussion

We investigated the POLST completion rate in patients with metastatic or recurrent cancer who were undergoing active anti-cancer therapy. When it was suggested by their physicians, 71.3% of patients agreed to prepare POLST, and most of them wanted to suspend CPR, artificial respiration, and hemodialysis. The POLST-completion group showed a higher proportion of highly educated patients and a better understanding of POLST. The participants answered that, typically, they would set up the EOL care plan of their own will. Their reasons for agreeing to or declining POLST were diverse.

More patients responded positively to POLST than expected by the researchers. To our knowledge, this is the first study that involved physicians’ active recommendation of POLST. On the basis of the high rate of POLST completion, we can infer that patients with metastatic or recurrent cancer were ready to begin a conversation about EOL, even during active treatment. However, the high acceptance rate could have been due to the “initial effect” of the policy. As this is the very beginning of POLST legislation in South Korea, the number of patients who had experienced multiple lines of chemotherapy and had considered their EOL in depth would have accumulated over time.

In our study, 67.1% of who completed POLST wanted to maintain chemotherapy in their EOL, which could be a result of misunderstanding. The Korean POLST is a single-step decision for patients. Nevertheless, in implementing the document, doctors’ decision involves two steps: initial time of POLST documentation and near EOL. Therefore, patients could be confused about the timing of POLST application. In our additional follow-up study, there were 35 of the total patients who wanted to maintain chemotherapy in their EOL were available to be asked again. After we had explained the situation thoroughly and asked whether they would still stand by their formal decision, 31 patients (88.6%) revised to not receiving chemotherapy. Based on this phenomenon, we suggest to revise Korean POLST to two-step approach.

Previous investigations from East Asian countries have shown that the decision to discontinue life-sustaining treatment is dominated by patients’ caregivers [4–7]. However, in our survey, most patients reported that they had made or would make their own decisions about EOL care. This phenomenon might have been owing to the study protocol—we did not give any prior notice about POLST and required participants to make their decision on the same day. Even though the patients had no time to discuss it with their families, more than half willingly completed POLST documentation on the same day.

It was their spouses that patients most often chose to have the discussion with, and doctors were preferred over offspring or siblings. This is consistent with the results of previous studies [8–10]. Regarding the reason for preparing POLST, a significant proportion of the participants (34.7%) answered that they did so at their doctor’s recommendation. Further, the wish to be treated by a familiar doctor was one of the major reasons for not using a hospice palliative center (37.8%). Therefore, the health care provider as well as familial support is important in patients’ critical decision-making process and EOL care.

Debate over the self-determination of EOL care has been actively conducted in the West, and POLST and AD originated from the concept of the “living will” that was first proposed by the Euthanasia Society of America in 1967 [11]. With this, lawyers and medical staff in the United States tried to provide legal grounds for living wills at the state level. However, the controversy over the “right to die” continued, and social discussion around EOL care expanded as several historical events occurred [12, 13]. In contrast to historical cases in the West, important cases in South Korea involved older subjects [14]. After several events and legal decisions, South Koreans started reviewing the social consensus on the following question: “What is ‘dying with dignity’?”

While the cases in the West mainly centered around the issue of “self-determination rights” of patients who entered a vegetative state at a younger age, the issues of the burden of support and financial difficulties of families have aroused more social sympathy in South Korea [14]. This is owing to differences in social values and cultural environment. Based on Confucianism, people in East Asian countries such as South Korea feel a high obligation to care for the elderly and provide familial support [15]. In many aspects, the care burden of the elderly has been re-established as a problem to be solved by the household, not by social security services [16]. The pressure and stress created by the issues of who should support and who should be supported are considerably high in South Korea [17, 18]. POLST and euthanasia can be a very dangerous scheme in this sociocultural environment. In some situations, someone may suspend his or her life-sustaining treatment to lessen the burden of the family. This kind of internal or external conflict can seriously undermine the original intent of POLST. In our study, 12.5% of the participants who agreed to complete POLST documentation said they decided to do so mainly because they wanted to ease the economic burden of their families. There was no significant difference when we compared the answers of the groups classified by monthly income, but further clarification is needed.

There has been some argument regarding when to prepare POLST in end-stage patients [19, 20]. In our study, a large percentage of participants answered that it would be desirable to prepare ACP when they were still young and healthy. Caregivers who accompanied the patients during the survey also expressed agreement with the withdrawal of futile life-sustaining treatment and asked whether they could fill out the document too. Although POLST in South Korea is restricted to end-stage patients, it is noteworthy that people are now more aware of the need to prepare for “dying well.”

The more patients understood life-sustaining treatment or POLST, the higher the completion rate was. A total of 66.7% of the completion group answered that they had more than a fair understanding of the system. Most of them got their information from traditional media such as the television or newspapers. A few weeks after the legislation, the South Korean POLST system began being promoted online. However, this is not the most effective way for older patients and those whose general condition is poor to access the relevant information. Physicians were the major source of information about EOL care to patients with cancer; thus, in-hospital education or counseling from medical staff could provide better assistance.

Our study had several limitations. First, the sample size was too small to achieve statistically significant *p*-values in many aspects. We also could not secure the diversity of cancer type because of the hospital system, making it difficult to represent the complete situation. Second, because different doctors recommended POLST in their own ways, the protocol could not be fully standardized. There was bias due to physicians’ diverse experience and rapport with patients. We assume that all study participants were well aware of the policy, but there might be discordance between doctors regarding eligibility criteria, length of instruction, and understanding of the process. The extraordinarily high rate of patients continuing with chemotherapy (67.1%) might be a result of misunderstanding. Third, because we did not collect detailed data about patients’ income and medical expense sources, we could not fully investigate the specific role of financial problems in POLST decision-making. The information on family members who were present at the completion day is also lacking, making it difficult to account for familial factors.

From social consensus to medical practice, there are still many issues that need to be addressed. Improving structural support for terminally ill patients, developing hospital-based assistance services, and providing education about the system would help yield positive outcomes. This study is meaningful in that it gathered opinions on the medical care of patients from the patients themselves, and not from their caregivers. We hope that the findings of this and subsequent studies can help people understand the POLST system and patients’ real attitudes toward EOL.

## Conclusion

With this study, we found that it is important to increase patients’ understanding of the policy in establishing end-of-life care. Better education and more efficient information sharing can be helpful in improving understanding of POLST in patients with cancer.

## Supplementary information

**Additional file 1: Table S1.** Responses to questions about end-of-life care.

**Additional file 2: Figure S1.** The relationship of advance care planning (ACP) with advance directives (AD) and Physician Orders for Life-Sustaining Treatment (POLST).

**Additional file 3.** Awareness and attitudes of patients with cancer toward physician orders for life-sustaining treatment(POLST).

## Data Availability

The datasets supporting the conclusions of this article are included within the article and its additional files.

## Abbreviations

ACP: Advance Care Plan
AD: Advance Directive
EOL: End of Life
POLST: Physician Orders for Life-Sustaining Treatment
