# Supplementary material for: Completion rate of physician orders for life-sustaining treatment for patients with metastatic or recurrent cancer: a preliminary, cross-sectional study
Source: BMC Palliat Care. 2019 Oct 22;18:84. doi: 10.1186/s12904-019-0475-9 (PMC6806497; doi:10.1186/s12904-019-0475-9)
Supplement: Supplementary file 1 — Additional file 1: Table S1. Responses to questions about end-of-life care. [file 12904_2019_475_MOESM1_ESM.docx]

**Additional file 1: Table S1. Responses to questions about end-of-life care**

| Question | Total  (N = 101) | POLST completion  (n = 72) | POLST non-completion  (n = 29) | *p*-value |
| --- | --- | --- | --- | --- |
| With whom do you mainly discuss your end-of-life care? No. (%) | | | | |
| No one | 51 (50.5%) | 39 (54.2%) | 12 (41.4%) |  |
| Spouse | 21 (20.8%) | 10 (13.9%) | 11 (37.9%) |  |
| Child | 13 (12.9%) | 6 (8.3%) | 7 (24.1%) |  |
| Sibling | 3 (3.0%) | 3 (4.2%) | 0 (0.0%) |  |
| Friends | 1 (1.0%) | 1 (1.4%) | 0 (0.0%) |  |
| Physician | 18 (17.8%) | 18 (25.0%) | 0 (0.0%) |  |
| Other | 1 (1.0%) | 1 (1.4%) | 0 (0.0%) |  |
| When do you think is the ideal time to discuss an advance care plan? No. (%) | | | | |
| When someone is young and healthy | 50 (49.5%) | 43 (59.7%) | 7 (24.1%) |  |
| Right after being diagnosed with cancer | 10 (9.9%) | 10 (13.9%) | 0 (0.0%) |  |
| When the disease progresses despite treatment | 21 (20.8%) | 12 (16.7%) | 9 (31.0%) |  |
| When everyday functioning is impossible owing to cancer-related symptoms | 16 (15.8%) | 5 (6.9%) | 11 (37.9%) |  |
| Other | 2 (2.0%) | 2 (2.8%) | 0 (0.0%) |  |
| Where do you think is the right place to meet your end of life? No. (%) | | | | |
| Home | 35 (34.7%) | 25 (34.7%) | 10 (34.5%) |  |
| Nursing home | 6 (5.9%) | 5 (6.9%) | 1 (3.4%) |  |
| Hospice care center | 23 (22.8%) | 21 (29.2%) | 2 (6.9%) |  |
| University hospital | 24 (23.8%) | 17 (23.6%) | 7 (24.1%) |  |
| Other | 11 (10.9%) | 5 (6.9%) | 6 (20.7%) |  |
|  |  |  |  |  |
|  | | | | |
