# Supplementary figures and images for: Completion rate of physician orders for life-sustaining treatment for patients with metastatic or recurrent cancer: a preliminary, cross-sectional study
Source: BMC Palliat Care. 2019 Oct 22;18:84. doi: 10.1186/s12904-019-0475-9 (PMC6806497; doi:10.1186/s12904-019-0475-9)

## Slide 1
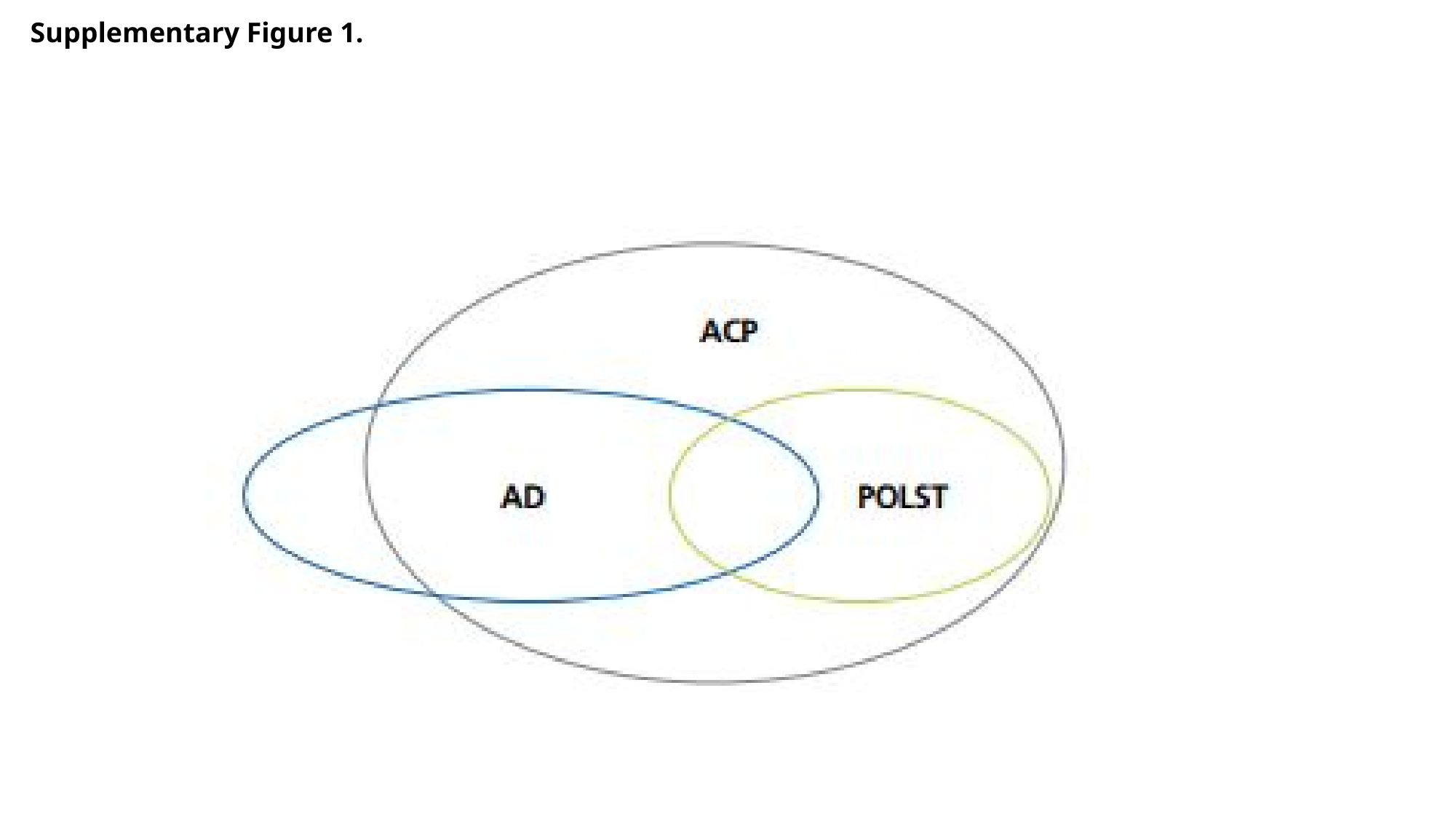

Supplementary Figure 1.
#

Supplement: Supplementary file 2 — Additional file 2: Figure S1. The relationship of advance care planning (ACP) with advance directives (AD) and Physician Orders for Life-Sustaining Treatment (POLST). [file 12904_2019_475_MOESM2_ESM.pptx]
