# Supplementary material for: Completion rate of physician orders for life-sustaining treatment for patients with metastatic or recurrent cancer: a preliminary, cross-sectional study
Source: BMC Palliat Care. 2019 Oct 22;18:84. doi: 10.1186/s12904-019-0475-9 (PMC6806497; doi:10.1186/s12904-019-0475-9)
Supplement: Supplementary file 3 — Additional file 3. Awareness and attitudes of patients with cancer toward physician orders for life-sustaining treatment(POLST). [file 12904_2019_475_MOESM3_ESM.docx]

**A-Type**

**Awareness and Attitudes of Patients with Cancer Toward Physician Orders for Life-Sustaining Treatment (POLST)**

This study is being conducted by the Korea University Anam Hospital Cancer Center as part of an investigation of awareness of and attitudes toward POLST. Your answers will be used for research purposes, and your personal information will be kept confidential. We appreciate your cooperation. Thank you.

| **Age** |  | **Gender** | M/F |
| --- | --- | --- | --- |
| **Religion** | None/Christianity/Catholicism/Buddhism/ Other: | | |
| **Education** | None/Primary school graduate/Middle school graduate/High school graduate/College graduate or above | | |
| **Primary caregiver** | None/Spouse/Children/Parents/Siblings/Other: | | |
| **Monthly income** | $100 or lower/$100–200/$200–300/$300 or more | | |

1. **Have you ever heard of “end-of-life care”? If yes, how much information do you have about it?**
2. I am very familiar with it
3. I am familiar with it
4. I have a fair understanding
5. I don’t know much about it
6. I don’t have any idea about it
7. **Where did you get your information about end-of-life care?**
8. From family
9. From friends and neighbors
10. Newspapers or television news
11. Internet
12. Physician
13. Other:
14. I have never heard of it
15. **Have you heard about the “medical decision-making law” or “POLST”? If yes, how much information do you have?**
16. I am very familiar with them
17. I am familiar with them
18. I have a fair understanding
19. I don’t know much about them
20. I don’t have any idea about them
21. **Where did you get information about the medical decision-making law or POLST?**
22. From family
23. From friends and neighbors
24. Newspapers or television news
25. Internet
26. Physician
27. Other:
28. I have never heard of them
29. **What is the major purpose of completing POLST today?**
30. To exercise my own will, not that of my caregiver
31. To ease the economic burden on my family
32. To avoid suffering from meaningless treatment
33. Because my doctor recommended it
34. Other:
35. **With whom did you mainly discuss your end-of-life care?**
36. No one (I decided on my own)
37. Spouse
38. Siblings
39. Children
40. Parents
41. Friends or neighbors
42. Physician
43. Other:
44. **When do you think is the optimal time for completing POLST?**
45. When someone is young and healthy
46. Right after being diagnosed with cancer
47. When the disease progresses despite treatment
48. When everyday life is impossible owing to cancer-related symptoms
49. Other:
50. **Where do you think is the right place to die?**
51. Home
52. Nursing home
53. Hospice care center
54. University hospital
55. Other:
56. **Would you prefer to use a hospice palliative care cancer if terminally ill?**
57. Yes, I would
58. No, I would not

**9-1. If your answer is “yes,” why?**

1. To receive professional treatment for pain and symptoms
2. To ease the economic burden of medical expenses
3. To reduce the burden on caregivers
4. To receive emotional and spiritual support
5. Other:

**9-2. If your answer is “no,” why?**

1. I don’t think it would be particularly helpful
2. I don’t have enough information about it
3. Because of the cost burden
4. I want to be treated by the doctor I’ve been seeing
5. Other:

**B-Type**

**Awareness and Attitude of Patients with Cancer Toward POLST**

This study is being conducted by the Korea University Anam Hospital Cancer Center as part of an investigation of awareness of and attitudes toward POLST. Your answers will be used for research purposes, and your personal information will be kept confidential. We appreciate your cooperation. Thank you.

| **Age** |  | **Gender** | M/F |
| --- | --- | --- | --- |
| **Religion** | None/Christianity/Catholicism/Buddhism/ Other: | | |
| **Education** | None/Primary school graduate/Middle school graduate/High school graduate/College graduate or higher | | |
| **Primary caregiver** | None/Spouse/Children/Parents/Siblings/Other: | | |
| **Monthly income** | $100 or lower/$100–200/$200–300/$300 or more | | |

1. **Have you ever heard of “end-of-life care”? If yes, how much information do you have about it?**
2. I am very familiar with it
3. I am familiar with it
4. I have a fair understanding
5. I don’t know much about it
6. I don’t have any idea about it
7. **Where did you get your information about end-of-life care?**
8. From family
9. From friends and neighbors
10. Newspapers or television news
11. Internet
12. Physician
13. Other:
14. I have never heard of it
15. **Have you heard about the “medical decision-making law” or “POLST”? If yes, how much information do you have?**
16. I am very familiar with them
17. I am familiar with them
18. I have a fair understanding
19. I don’t know much about them
20. I don’t have any idea about them
21. **Where did you get your information about the medical decision-making law or POLST?**
22. From family
23. From friends and neighbors
24. Newspapers or television news
25. Internet
26. Physician
27. Other:
28. I have never heard of them
29. **What is the major reason for not completing POLST today?**
30. Need more time by myself to think about it
31. Need to discuss it further with family
32. Lack of understanding of the system
33. I don’t think it’s the right time yet
34. Other:
35. **With whom do you plan to discuss your end-of-life care?**
36. No one (I will decide on my own)
37. Spouse
38. Siblings
39. Children
40. Parents
41. Friends or neighbors
42. Physician
43. Other:
44. **When do you think is the optimal time for completing POLST?**
45. When someone is young and healthy
46. Right after being diagnosed with cancer
47. When the disease progresses despite treatment
48. When everyday life is impossible owing to cancer-related symptoms
49. Other:
50. **Where do you think is the right place to die?**
51. Home
52. Nursing home
53. Hospice care center
54. University hospital
55. Other:
56. **Would you prefer to use hospice palliative care cancer when terminally ill?**
57. Yes, I would
58. No, I would not

**9-1. If your answer is “yes,” why?**

1. To receive professional treatment for pain and symptoms
2. To ease the economic burden of medical expenses
3. To reduce the burden on caregivers
4. To receive emotional and spiritual support
5. Other:

**9-2. If your answer is “no,” why?**

1. I don’t think it would be particularly helpful
2. I don’t have enough information about it
3. Because of the cost burden
4. I want to be treated by the doctor I’ve been seeing
5. Other:
